# Moderated mediation role of ethnicity on natural skin care products purchasing intention model among multicultural consumers

**DOI:** 10.1371/journal.pone.0300376

**Published:** 2024-03-21

**Authors:** Ahmed Abdulkareem Najm, Sarah Abdulkareem Salih, Shazrul Fazry, Douglas Law

**Affiliations:** 1 Department of Food Sciences, Faculty of Science and Technology, Universiti Kebangsaan Malaysia, Bangi, Selangor, Malaysia; 2 Department of Architecture, Faculty of Design and Architecture, Universiti Putra Malaysia, Serdang, Selangor, Malaysia; 3 Tasik Chini Research Center, Faculty of Science and Technology, Universiti Kebangsaan Malaysia, Bangi, Selangor, Malaysia; 4 Faculty of Health and Life Sciences, Inti International University, Nilai, Negeri Sembilan, Malaysia; Sichuan Agricultural University, CHINA

## Abstract

The trends for sustainable lifestyle and marketing motivated natural product consumption, such as natural skin care products (NSCPs). Different personal, environmental, and sociocultural factors influence purchase intention (PI) for NSCPs. However, there is a lack of evidence on the role of consumers’ ethnicity in the PI model. The present study investigated the moderated mediation role of ethnicity in the relationship between related factors, including environmental concern, subjective norms, health factor, Halal certificate, packaging design, past experience factor, price factor, and PI mediated by personal attitude. A web-based survey was utilized to capture quantitative data from a random sample of 330 multicultural consumer group participants. The results of the study indicated that consumers’ ethnicity substantially moderated the mediation effect of personal attitude in the relationships between subjective norms, health factor, Halal certificate, packaging design, past experience factor, price factor, and PI in the model. The findings contributed to understanding of the factors that influenced the PI of consumers from diverse sociocultural contexts in the market for natural products. It contributed directly to natural product marketing and industry.

## 1. Introduction

Natural products have recently gained an increase interest in the cosmetics market due to their positive impact on climate and health [1–3]. Natural products, such as plant and animal extracts have been broadly used for overall body care and to treat various health issues, including skin issues [2, 4]. Natural care products have been a synonym for sustainable, green, organic, or healthy products, which have a reduced negative effect on humans and the environment. This rising awareness of the environmental impact and sustainability has led to the development of a new approach in purchasing natural skin care products (NSCPs) [3, 5]. Purchasing concerns for these products could promote environmental sustainability and a healthy lifestyle, and reduce environmental deterioration [4, 6]. Natural products are also widely used in cosmetic formulations, including cosmetics and personal care products [7]. Recently, skin care has become a routine daily procedure in every household by individuals of different sociodemographic backgrounds [8, 9]. Today, there is a wide variety of skin care products in the global market, some of which are natural and some synthetic. NSCPs are generally the common choice for consumers nowadays [10].

Recently, several studies investigated on multi-correlation and mediating effect between related internal and social factors (social, physical, health, and environmental factors) and purchase behavior [11–14]. However, only a few studies examined the role of sociocultural variables and social, personal, and physical factors as moderated mediators for consumers’ purchasing behavior. Sociodemographic variables, such as gender, play a significant role in the framework of green and natural purchase activities [15, 16]. Nevertheless, most existing evidence neglected the moderated mediation role of sociocultural variable, such as ethnic background in the framework of natural purchase intention (PI). In social science theory and research, the moderation effect of social and sociodemographic variables had been shown [17, 18]. few pieces of evidence from psychological sciences also showed the moderated mediation effect of ethnicity [19, 20].

On the other hand, one of the focal points of marketing research and consumer research is the multicultural market [21]. The multicultural market involves marketing to more than one ethnicity of consumers. It takes advantage of the ethnic groups to communicate with consumers [21]. A significant example of a multicultural market is the Asian market, particularly the Malaysian market, whereby Malaysia is a multicultural society and its market consists of cross-cultural consumers. Since ancient times, Asian nations have recognized and used the medicinal properties of herbs, plants, animals, and aquatic extracts [22, 23]. They still utilized traditional and natural sources for medication and personal care products and were aware of the safe effects of such products [23]. They are aware of the safe effects of such products [22, 23]. Malaysian consumers are also looking at the natural components of modern personal care products. Recently, they showed an increased interest in these products, with about 10% of annual purchase rate in 2020, indicating an exciting increase in PI for natural products among consumers in Malaysia [5]. However, the Ministry of Health Malaysia reported that the multicultural market in Malaysia was overwhelmed with chemical care products containing toxic ingredients that negatively impact consumers’ health [24]. A significant number of Malaysian consumers keep on purchasing chemical care products even though they understand the profound negative impact of these products [25]. The reason behind these conflicting percentages in natural and chemical care product purchasing is still incomprehensible [26].

Furthermore, Malaysia has three significant ethnicities: Malays, Chinese, and Indians. In Malaysia, ethnicity is an important characteristic that predicts individuals’ cultural, religious, social class, and political backgrounds [27]. Each ethnic group is distinguished by subjective norms, attitudes, behavior, and preferences. Therefore, ethnicity plays a critical role in peoples’ behavior. Evidence from other frameworks than consumers’ PI had shown the significant moderation model of ethnicity in Malaysia as a multicultural community, such as the ethnocentrism and social capital framework [28], political socialization [29], and security framework [30]. However, moderated mediation role of ethnicity on natural care product PI through related personal, social, and physical environmental factors is still unclear in the multicultural (Malaysian) context. Consequently, this study constructed a novel moderated mediation model in correlation between PI and related factors to introduce theoretical bases for multicultural marketers to increase NSCPs purchases and enhance consumers’ behavior.

Therefore, this study aimed (1) at determining the wide range of intention predictors to purchase NSCPs among multicultural customers (2). Furthermore, this study investigated the potential mediating effect of personal attitude between related environmental, social, and personal factors and multicultural consumers’ intention to purchase NSCPs (3). The study also examined the potential moderated effect of ethnicity on personal attitude mediation role among related environmental, social, and personal factors and multicultural consumers’ intention to purchase NSCPs. Consequently, this study suggested a moderated mediation model for ethnicity interaction with associated factors on natural care products PI to provide policymakers, and local and international marketers with a theoretical strategy for enhancing natural care product purchases in the multicultural market. It seeks to understand buying intention for natural products in the multicultural market from a new perspective, including cultural and racial differences, and various broad factors of buying attitudes.

## 2. Literature review

### 2.1. PI toward NSCPs

Skin care products are widely used to promote skin appearance, enhance its integrity, and relieve its issues and disorders. They can include various types of creams and emollients, cleansers, toners, serums, exfoliators, lotions, and other cosmetics [31]. Natural skin care has become the daily regimen of choice for consumers in a variety of settings due to its positive efficacy and fewer side effects on health [32]. Individuals have become more concerned about skin care products ingredients and the side effects of conventional products, which may influence their purchasing decisions [33]. Compared to synthetic skin care products, NSCPs are safer due to their natural ingredients. NSCPs comprised plant or animal-derived raw materials, and are abundant in fine particles and minerals that serve a variety of functions for skin health. Since the skin is a fast-absorbing surface, the ingredients contained in anything it is exposed to can be slowly absorbed into the bloodstream [34, 35]. Therefore, NSCPs work to avoid adverse health effects and provide more protection [12, 32]. Additionally, NSCPs contribute effectively to sustainability and environmental protection [34]. Therefore, consumers are acute to use safer alternatives on conventional skin care products, such as natural products. The ingredients contained within synthetic skin care products can also cause side effects or allergic reactions on the skin. On the contrary, NSCPs are free of irritants and are beneficial to the skin [36].

On the other hand, PI refers to personal willingness of consumers to purchase a particular product that predicts the possible purchasing intention of consumers in the consumption market [37–39]. Therefore, PI toward NSCP refers to the consumer’s willingness to purchase NSCPs more than other chemical products. PI is usually a dependent variable influenced by several external, internal, and sociocultural factors [40]. Evidence suggested that diverse social and environmental factors might have a direct effect on customers’ inclination to purchase NSCPs, predicting their intention in the natural skin care industry [12, 20, 41–43]. Moreover, evidence showed a possible mediator effect of internal factors, such as personal norms, personal values, and personal attitudes [16].

Existing studies also examined the moderator effect of sociodemographic factors, such as educational level [5], and external factors, such as media exposure. In the Malaysian context, there is a great demand for international and local natural or synthetic personal care products the sales of both global and local skin care, and cosmetic products reached RM2.64bn (USD600 million) in 2020 [5]. In Malaysia, different cosmetic products are submitted to the Control of Drugs and Cosmetics Regulations 1984 (Sale of Drugs Act 1952) to ensure safety, provide low-risk products, and avoid prohibited ingredients [24]. The cosmetic manufacturing regulations in Malaysia are similar to the cosmetic regulatory procedure in Southeast Asian countries (ASEAN), and other countries in Europe and the USA [24]. Recently, skin care market has shown an increased interest in natural skin products made from natural ingredients [5, 26] and for cosmetic Halal products that are derived from safe ingredients for consumers and unadulterated with filth [7, 26]. However, the Malaysian natural skin care market is still fresh. Although it is significantly growing, there is a lack of research on consumers’ PI and related factors in the natural skin care Malaysian market context. Moreover, the Malaysian market is multicultural, whereby several interacted factors can influence. This calls for further studies to investigate consumers’ PI for NSCPs in the Malaysian market.

### 2.2. Theoretical bases and hypothesis formulation

The theoretical foundations of this study were built based on existing literature and PI research and theories, such as the value-belief-norm theory (VBN), consumption value theory (TCV), and planned behavior theory (TPB), using the moderated mediation model [18]. These notions are essential for comprehending an individual’s behavior. The VBN theory suggested that an individual’s values and concerns influenced one’s behavior or activity in an environmentalist context, which indicated the individual’s environmental behavior. More specifically, it indicated that individuals’ environmental concerns could affect PI through personal values or personal attitudes in the natural products market [44, 45]. This theory is usually applied to understand environmentally friendly attitudes. Therefore, it explains consumers’ green purchase behavior or intention based on their values and concerns, such as environmental concerns [24]. Existing studies based on VBN also supported the relationship between environmental concerns and purchase intention through personal attitudes in the PI model [16, 24, 26]. Therefore, VBN and the existing body of knowledge were used in theoretical basis of the current study to suggest the direct relationship between environmental concerns and personal attitude, and natural skincare product PI (H1 and H8). Additionally, they were used to set indirect (mediation effect) personal attitudes in the relationship between related factors, such as environmental concerns and natural skincare product (H9).

Ajzen (1991) developed a psychological theory called TPB, whereby he believed that an individual’s behavioral intentions, such as PI were influenced by the personal attitude variable. It provided an understanding of individuals’ PIs through their cognitive attributes, including personal attitudes, personal health concerns, and subjective norms. It examined consumers’ purchasing intention toward a specific good as a planned behavior based on the consumers’ personal attitudes and preferences [46]. The TPB is extensively utilized to investigate vast social and behavioral research domains [5, 47]. Existing studies also used TPB in behavioral research on natural product consumption. Additionally, a few studies based on TPB indicated the relationship between subjective norms, health concerns, personal attitudes, and PI in the natural purchase intentions model. TPB was used to test the relationship between health and environmental concerns and PI toward green cosmetics moderated through attitude and consumer demographic factors [16]. TPB was also used to test whether personal attitudes and subjective norms were significant predictors of natural product PI [31]. Therefore, TPB and existing literature were used to set the direct and indirect (mediation effect) personal attitudes in the relationship between the subject norms, health concerns, and natural skincare product PI (H2, H3, and H9).

The TCV is a multi-dimensional approach, suggesting that social, conditional, environmental, functional, and epistemic values are critical predictors of consumers’ behavior in the market [48]. It explains how and why consumers choose to purchase or not to buy a specific product or brand over another, based on a different set of considerations. Therefore, TCV helps to explain the possible relationship between various personal values, including environmental concern, subjective norms, external factors (e.g., product price and past experience), and PI through a mediator or moderator variable [33, 34, 49, 50]. Furthermore, TCV helps to predict natural product PI through the third variable (social-cultural factors), such as family size [1] and ethnic background [51]. Regarding ethnic background, consumers from different ethnic backgrounds might have different purchase behavior patterns driven by other social and environmental factors [51]. Therefore, TCV and the existing studies were used to set the direct relationship between related factors, such as environmental concerns, subjective norms, and other external factors (product price and past experience) and PI (H1 –H7). The three theories, VBN, TPB, and TCV, along with existing literature were also used to set the theoretical basis of the current study. This was done by suggesting the indirect (mediation and moderation effects) personal attitudes and consumers’ sociocultural variables in the relationship between environmental concerns, health concerns, subjective norms, and external factors (e.g., product price, past experience, Halal certificate concerns, and packaging design), and natural skincare product PI (H10).

**Fig 1:**
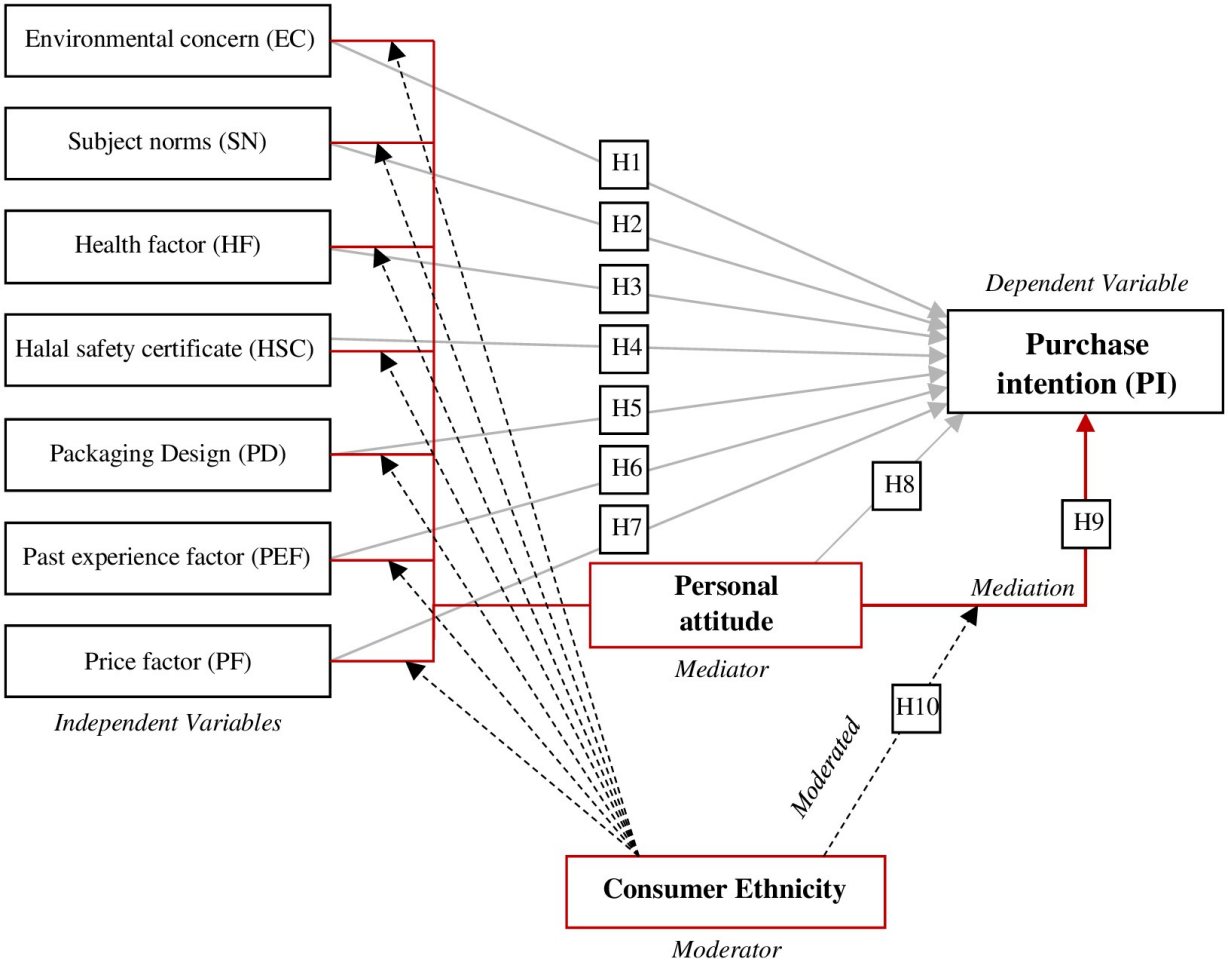
The conceptual framework of the moderated (ethnicity) mediation (personal attitude) effect between related factors (IVs) and PI (DV).

However, little is known about the role of personal attitudes and sociocultural variables in the relationship between personal experience, health concerns, product external factors, and PI in the natural products model. Moreover, in Malaysia, there is a shortage of studies on the moderation and mediation role of consumer’s ethnic background in the natural consumption model. Taking into account these gaps in the existing literature and the mentioned theories utilizing the methodology of Preacher et al. [18], the conceptual framework of the ethnicity (as a sociocultural factor) moderated mediation role is devised (see Fig 1).

#### 2.2.1. Environmental concern and PI toward NSCPs

Environmental concern is one of the critical factors affecting personal PI [34, 52]. Several existing studies had focused on internal factors, such as environmental attitude, environmental consciousness, or environmental belief [53], which represented the effect of environmental concern factors on consumers’ PI for NSCPs [5]. Environmental concern refers to an individual’s belief that human activities and behavior affect the biophysical environment. Certain levels of environmental concern are essential in environment-friendly and sustainability initiatives [54]. Environmental concern is vital to motivate individuals to participate in sustainable natural consumption [55]. A few Malaysian studies found that environmental concern positivity affected the intention of multicultural consumers to purchase NSCPs [5]. However, consumers’ attitudes, backgrounds, and sociodemographic might have a significant role in the relationship between related environmental factors and consumers’ PI [12, 34, 56]. Moreover, there is a need for evidence on the relationship between environmental concern and PI among multicultural consumers in the natural skin care markets. Therefore, the following is hypothesized:

Hypothesis 1: Environmental concern positively affects multicultural consumers’ intention to purchase NSCPs.

#### 2.2.2. Subject norms and PI toward NSCPs

Subjective norm indicates the effect of family and community social aspects on consumers’ intention and behavior to purchase a particular product [52]. Existing studies considered various factors of subjective norms, such as family, friends, community, advertisements, social media, and celebrity effects [52, 57, 58]. Furthermore, some existing research confirmed that subjective norms was a significant variable influencing sustainable skin care consumption; it encouraged consumers to purchase NSCPs [59]. Liobikienė and Bernatonienė suggested to conduct future research on the influence of subjective norms on PI in the community cultural variable and cross-cultural community contexts [33]. However, Kumar confirms that subject norms has an insignificantly direct impact on the PI for environmentally sustainable products [59]. Research from multicultural communities indicates that consumers’ academic background (as a sociodemographic factor) influences the relationship between subject norms and the PI model [5]. However, they did not find a direct effect of consumers’ subjective norms on PI toward green skin care products. Consequently, consumers’ sociocultural and demographic variables could significantly affect the relationship between subjective norms and PI. Moreover, there is a lack of studies on the relationship between health factors and PI in the moderation mediation model among multicultural consumers. Therefore, in light of the existing literature and theories, the following hypothesis is proposed.

Hypothesis 2: Subjective norms positively affect multicultural consumers’ intention to purchase NSCPs (see Fig 1).

#### 2.2.3. Health factor and PI toward NSCPs

Health factor refers to individuals’ health consciousness, health concern, or health awareness, which is the degree of care about their health [60]. Health consciousness factors drive individuals to engage in healthy behavior to maintain a healthy life [61]. If consumers have a higher level of health consciousness, they are more likely to consider product safety and contribute to purchasing sustainable and natural products [53]. Therefore, health factor is a possible factor positively affecting PI for NSCPs [61]. Research from Malaysia also showed that health factors, such as health value and health concerns, potentially affected organic product consumption [31]. Nonetheless, there is a shortage of research on health factor in the moderated mediation and consumers’ PI for NSCPs, particularly among multicultural consumers. Moreover, existing studies showed disagreement on the critical factor affecting consumers’ PI in the skin care market. Based on existing literature, the following is hypothesized.

Hypothesis 3: Health factor significantly affects multicultural consumers’ intention to NSCPs.

#### 2.2.4. Halal safety certificate and PI toward NSCPs

Existing studies indicated that the safety certificate is an external factor influencing consumers’ intention to buy selected products [56]. Safety certificate refers to availability of the safety certificate logo on the product packaging, indicating successful testing process of the product for the required qualifications, such as the Halal safety certificate by the Halal Management Division in Malaysia [62]. Recent evidence in natural product PI studies showed that safety certificate was a significant variable contributing to consumers’ PI to the certified product [63]. Halal safety certificate refers to presence of the Halal emblem on products, which denotes a testing procedure based on the qualification of Halal products to ensure compliance with Halal Islamic consumers’ product requirements [62]. A few Southeast Asian studies indicated that Halal safety certificate and attitudes toward Halal products substantially affected the buying intention for cosmetic products [62, 64, 65]. However, Ahmad et al. showed that knowledge of the products’ Halal logo does not significantly affect consumer intention in purchasing cosmetic products [62]. Contrarily, Nordin et al. and Aziz et al. confirmed that producing Halal products attracted the interest of consumers globally in the Halal beauty industry, which provided safe products unadulterated with filth. In addition, most of the existing studies on Halal safety certificate focus on purchase behaviors of cosmetic and food products from a general perspective However, blending harmful substances and fats into cosmetics and natural products has been indicated, which might influence the purchasing intention toward NSCPs. Additionally, most of the existing studies on Halal safety certificate focused on purchase behavior of cosmetics and food products from a general perspective [62, 64]. Therefore, there is a need to study the Halal safety certificate in the PI model of NSCPs; hence, the following hypothesis is developed:

Hypothesis 4: Halal safety certificate significantly affects multicultural consumers’ intention to purchase NSCPs.

#### 2.2.5. Packaging design and PI toward NSCPs

Existing studies also included the packaging design of natural products as an external factor affecting the natural product PI model [41, 66]. Packaging design refers to the parameters of product packages that affect buying intention for certain goods and items, such as NSCPs [67]. Packaging design parameters, such as color, material, and texture stimulate consumers’ senses and contribute to PI [41, 67–69]. The innovative design of product packaging is also a significant determinant of PI toward NSCPs [70]. It could indirectly affect PI through the individuals’ attitudes and environmental factors [41]. In the multicultural market, however, there are insufficient studies revealing the relationship between the packaging design of NSCPs and consumers’ PI in the moderated mediation model. Consequently, the hypothesis is the following:

Hypothesis 5: Packaging design significantly affects multicultural consumers’ intention to purchase NSCPs.

#### 2.2.6. Price factor, past experience factor, and PI toward NSCPs

Furthermore, existing literature highlighted the products price and consumers’ past experience factors as possible factors influencing consumers’ intention to purchase the targeted products, such as NSCPs [53, 71]. Based on Boztepe price factor is one of the most relevant external factors influencing the PI in the natural product industry [71]. Liobikienė et al. believe that a high price level of natural products is the main barrier to purchasing intention toward organic cosmetics and NSCPs [33]. However, Chekima et al. mentioned that price level has no significant impact on green consumerism [72]. Past experience in purchasing skin care products also plays a critical role in cosmetic marketing [73]. However, Kumudhini and Kumaran only focused on the impact of past consumer experience on the intention to purchase organic cosmetics in the Sri Lanka cosmetic market [73]. Yet, there is a lack of study on the past experience factor and price factor in the PI model for NSCPs among multicultural consumers. Hence, the following hypothesis is developed:

Hypothesis 6: Past experience factor significantly affects multicultural consumers’ intention to purchase local NSCPs.
Hypothesis 7: Price factor significantly affects multicultural consumers’ intention to purchase NSCPs.

#### 2.2.7. Mediation effect of personal attitude toward NSCPs

Existing studies have indicated that personal attitude is an internal factor (personal factor) affecting PI [34, 74]. Personal attitude refers to the urge to engage in or respond positively or negatively to a particular item, activity, or conviction, which leads to an individual’s behaviour. Individuals with a positive personal attitude towards engagement in the behavior are more likely to engage in the conduct or positively respond to it [34]. For instance, a positive attitude towards NSCPs substantially predicts the intention to purchase NSCPs [57, 75]. As a result, personal attitude toward NSCPs refers to a personal desire to use or not utilize NSCPs, which results in specific purchase intention [34]. Evidence from multicultural markets also demonstrates a significant positive association between one’s attitude toward natural personal care goods and their PI [26, 31]. personal attitude to natural care products might also influence consumers’ attention to the local market [12].

Recent studies on PI assume that different sets of environmental or personal factors mediate the relationships between the other factors and PI [24, 35]. For example, Choi et al. examine the mediating effect of personal norms between environmental beliefs and consumers’ green decisions [76]. Mamun et al. reveal that PI mediated the impact of environmental factors and personal attitude on Malaysians’ purchasing intention of green products [5]. Jaini et al. investigate the mediating effect of environmental belief in the relationship between subjective norms and social values in the context of the cosmetics market [26]. Besides, sociodemographic factors such as education are also observed as mediators in the health outcome model [77] and the psychology model [78]. Peyvandi et al. confirmed that maternal education mediated the most significant percentage of racial disparity among cardiovascular disease patients [77]. Income level is also a possible mediator factor in the mediation model [78]. Nevertheless, there has been little research on the role of personal attitude as a mediator in the moderated mediation model for the relationships between predictor variables and PI toward NSCPs among multicultural consumers. As a result, the hypotheses are as follows:

Hypothesis 8: Personal attitude significantly affects multicultural consumers’ intention to purchase NSCPs.
Hypothesis 9: Personal attitude significantly mediates the relationship between related factors (environmental concern, subject norms, health factor, Halal safety certificate, DP, past experience factor, and price factor) and intention to purchase NSCPs among multicultural consumers.

#### 2.2.8. Moderated mediation role of ethnicity on the PI model

Furthermore, existing research also investigates the moderator role of external factors such as brand and brand trust in the natural PI model [13]. Media exposure (external factor) was also examined as a moderated mediation variable in the relationship between gender and green PI in the Chinese market [15]. A recent study among multicultural consumers conducted by Najm et al. finds that the safety certificate (external factor) on NSCPs significantly moderated mediation in the PI model [41]. However, Chekima et al. confirm that price level (external factor) has no moderating effect on the relationship between product quality and green PI [72]. Thus, there is a disagreement on the moderation interaction on the intention to purchase natural products.

Yang and Zhao explore the moderator role of household income (sociodemographic variable) between personal attitude and PI for energy-efficient equipment in China [79]. Sánchez-Torres et al. also find strong evidence of the moderating effect of educational level and socioeconomic status on the electronic PI [42]. Another study from China reveals the moderated mediation effect of gender within the framework of green purchasing intention [16]. Besides, Cooray et al. suggest that the ethnicity of Sri Lankan females has a critical moderating effect on the relationships between brand names and purchase decisions [80]. A study from the USA on multicultural participants demonstrates that ethnicity is a moderated mediated factor in the correlation between acculturative stress and attitudes [19]. Nonetheless, there is a shortage of evidence on the moderated mediation of sociocultural variables such as ethnicity on the natural products PI model, especially in the multicultural Malaysian context. Malaysia is a multicultural society where ethnicity is expected to play a significant role in individuals’ purchase behaviors and preferences. Besides, people from a particular ethnicity might have certain environmental, social, and personal beliefs that vary from those of other ethnicities. Therefore, ethnic differences emerge based on social, cultural, and environmental beliefs. Malaysia consists of three significant ethnicities; the Malay group represents about 69.6%, 22.6% Chinese, 6.8% Indian, and 1% other ethnicities. Therefore, in line with the existing study and to fill the literature gap on the moderated mediation mechanism in the PI model, this study examines the possible moderated mediation role of ethnicity in the natural skin care PI model (see Fig 1). The hypotheses are as follows:

Hypothesis 10: The ethnicity of multicultural consumers significantly moderates the mediation effect of personal attitude between related factors (environmental concern, subject norms, health factor, Halal safety certificate, DP, past experience factor, and price factor) and the intention to purchase NSCPs.

## 3. Methodology

A web-based questionnaire survey is used to test the proposed theoretical bases based on Creswell’s recommendations [81]. In line with the objective of the present research, multicultural consumers from Malaysia of the three main ethnicities, Malay, Chinese, and Indian, aged 18 and over, are the targeted population. The study sample is selected using a non-probability convenient sampling approach. The sample size is identified based on Creswell [81] approach to having a minimum of ten times responses as the number of questionnaire items (25 X 10 = 250 minimum) to be accepted in multivariate data analysis. Therefore, 330 participants who participated in the online survey were received in three months, from 30th April to 1st July 2022. The data and questionnaire responses were gathered utilizing an online survey platform known as Google Forms. The questionnaire survey was disseminated using popular social media channels, such as WhatsApp and e-mails. At the outset, the participants were questioned regarding their purchasing habits concerning NSCPs. This screening question aimed at ascertaining that the individuals involved possess a high level of expertise in procuring NSCPs [41]. The present study data is collected as part and continue of another study published by Najm et al. [41]. The preliminary data has been provided in the attached supplementary files.

### 3.1. Items of the questionnaire survey

Items and scales of the questionnaire were modified from prior research with minimal modifications to reflect the context of the present study. Based on the study variables, the questionnaire was separated into three parts Table 1. The first part includes questions on sociodemographics (participant variables), including gender, age groups, ethnicity, educational level, occupation, marital status, and monthly household income adapted [5, 12, 26]. It also consists of a question on the sociocultural variable of participants’ ethnicity (moderator) adapted from Jaini et al. [26], including Malays, Chinese, and Indians. The second part of the questionnaire consists of three questions on consumers’ intention to purchase natural skincare products (independent variable) adapted from Najm et al. [41] and Zollo et al. [52]. The third part of the questionnaire includes the related independent variables that could affect multicultural consumers’ intention to purchase NSCPs and the mediator variable. The personal attitude (mediator) is measured using three items from two studies Mamun et al. [5] and Khan and Salim [82], Table 1. Three items derived from Mamun et al. [5] and Zollo et al. [52] are used to measure environmental concern. Three items adapted from Jaini et al. [26] and Khan and Salim [82] are used to evaluate subject norms. Two items modified from Khan and Salim [82] measure health factors. Meanwhile, the Halal safety certificate is calculated using three items adapted from Ahmad et al. [62]. Packaging design is assessed using four items designed by Imiru [67] and Prabowo and Aji [70]. Past experience factor is measured with a two-item scale proposed by Khan and Salim [82]. Furthermore, price factor is measured by two items designed by Cadar et al. [12]. The second and third sections of the questionnaires utilize a 5-point Likert scale ranging from 1 = strongly disagree to 5 = strongly agree [41], Table 1.

**Table 1:** FL, CA, CR, and AVE of the questionnaire.

Items		Description	FL (>.70)	CA (>.70)	AVE (>.50)	CR (>.60)	Source
**Environmental concern (EC)**	EC1	I prefer natural skincare products that have been prepared in an environmentally friendly way.	.806	.902	.667	.857	[5]
	EC2	I prefer to buy natural skincare products because they are environmentally safe.	.838	-	-	-	[5]
	EC3	I will buy natural skincare products instead of conventional products to contribute to a healthy environment.	.807	-	-	-	[52]
**Subjective norms (SN)**	SN1	I prefer to buy natural skincare products that recommend by my family members or friends	.790	.846	.646	.845	[26]
	SN2	Advertisement on natural skincare products motivates me to buy the product	.829	-	-	-	[26]
	SN3	Celebrity endorsement of natural skincare products motivates me to buy the products	.793	-	-	-	[82]
**Health factor (HF)**	HF1	I am concerned about my health when I buy any products I use in my daily life, including skincare products	.785	.849	.581	.734	[82]
	HF2	I am concerned about my health in my daily life behaviors	.739	-	-	-	[82]
**Halal safety certificate (HSC)**	SC1	I prefer to buy Halal-certified skin products	.866	.909	.695	.872	[62]
	SC2	I prefer to buy Halal-certified skin products because they are safe and reliable	.851	-	-	-	[62]
	SC3	Natural skincare products made in Malaysia are necessarily Halal	.782	-	-	-	[62]
**Packaging Design (PD)**	PD-1	The innovative packaging design of natural skincare products motivates me to buy the product.	.782	.828	.643	.900	[70]
	PD-2	The color of the packaging of natural skincare products motivates me to buy these products.	.767	-	-	-	[70]
	PD-3	The attractive color combination of natural skincare products’ packaging motivates me to buy these products.	.777	-	-	-	[70]
	PD-4	The material used for packaging natural skincare products motivates me to buy these products.	.866	-	-	-	[67]
**Past experience factor (PEF)**	PEF1	I like to experiment with new natural skincare products	.778	.834	.607	.775	[82]
	PEF2	I am open to trying something new in the cosmetic market	.781	-	-	-	[82]
**Price factor (PF)**	PF1	The price range of the product motivates me to buy the natural skincare products	.706	.701	.501	.667	[12]
	PF2	Advantages offered, such as promotions and discounts, motivate me to buy the natural skincare products	.710	-	-	-	[12]
**Personal attitude (PA)**	PA1	I have a positive attitude toward natural skincare products.	.813	.900	.610	.824	[5]
	PA2	I prefer using natural skincare products more than conventional skincare products because they are safe.	.797	-	-	-	[5]
	PA3	I look for herbal and natural ingredients in skincare products over chemical ingredients.	.732	-	-	-	[82]
**Purchase intention (PI)**	PI1	I intend to purchase natural skincare products than conventional (traditional chemical) skincare products	.818	.782	.551	.785	[41]
	PI2	When I have a choice between two equal skincare products, I will purchase the natural one	.700	-	-	-	[41, 52]
	PI3	I intend to buy natural skincare products in the future	.703	-	-	-	[41, 52]

### 3.2. Content and convergent validity

The questionnaire items were validated using the Content Validity Index (CVI) and Average Variance Extracted (AVE), as suggested by Shrotryia and Dhanda [83]. The content of the questionnaire items was reviewed by four experts (three associate professors and one senior lecturer) from three international universities. Overall, three experts are adequate to verify the content validity of any questionnaire [83]. The CVI for overall relevance, readability, and transparency is greater than 0.78 see Table 2. This result indicates that the four experts agreed that the survey questionnaire questions were relevant and valid [83]. The researcher also modifies the format of the questionnaire based on the suggestions of the experts.

**Table 2:** I-CVI for questionnaires by four experts.

	Items	Expert 1	Expert 2	Expert 3	Expert 4	No in agreement	I-CVI
**Relevantly**	Section A	3	4	4	4	4	1 (4/4)
	Section B	3	4	3	4	4	1
	Section C	4	4	3	3	4	1
**Readability**	Section A	3	4	4	4	4	1
	Section B	3	4	3	4	4	1
	Section C	4	4	4	4	4	1
**Clarity**	Section A	3	4	4	4	4	1
	Section B	3	4	4	4	4	1
	Section C	4	4	4	4	4	1

**Note:** 1 = not relevant, 2 = somewhat relevant, 3 = quite relevant, 4 = highly relevant.

3 and 4 represent an agreement on the items.

**Source:** Author’s data analysis.

The AVE is the quantity of variance a construct (questionnaire item) can explain in its respective indicators [84]. AVE should be greater than or equal to 0.50 to indicate substantial variance with individual construct items [84]. Table 1 demonstrates that all questionnaire items had AVE values greater than 0.50, confirming their convergent validity.

### 3.3. Reliability test and Common Method Bias (CMB)

Table 1 shows the factor loading, validity, and reliability tests on the study protocols and questionnaire items [85]. Reliability was assessed using the factor loading (FL), composite reliability (CR), and internal consistency Cronbach’s alpha (CA). The FL, CR, and CA were calculated with the aid of SPSS 25 and Excel 2019. The instrumentation stability, dependability, consistency, and reproducibility levels are referred to as reliability [85, 86]. Due to low FL (.70), one item from each of the subject norms, past experience factor, and packaging design and two items from price factor are deleted. As a result, the values of each FL, CA, and CR exceed the threshold value, confirming the reliability of the survey questions see Table 1. Based on Bolarinwa [85], to obtain an acceptable level of reliability, FL and CA must be larger than 0.70, and CR must be larger than 0.60. In addition, the results of internal consistency reliability indicated that the total CA for all questionnaire items was 0.93. Factor analysis was also employed for CMB, which explained 42.84% of the variance per single component regarding all items.

### 3.4. Ethical statement

In the country where the study was conducted, ethical approval is not required for this form of research (social surveys). Participation in this survey was entirely voluntary. Before applying the survey or any related procedure, all the participants gave their informed consent.

### 3.5. Data analysis method

The total data was analyzed in two stages. Firstly, the validity and reliability stage was conducted to guarantee internal consistency, reliability, factor loading, and content validity. Secondly, the study theoretical foundation was evaluated regarding its ability to anticipate outcomes [24, 26]. The sociodemographic variables of the subjects are examined using straightforward frequency statistics (control variables). All study variables, including sociodemographic variables, PI (the dependent variable), personal attitude (the mediator), ethnicity (the moderator), and associated factors (the independent variables), are analyzed using descriptive statistics. A bivariate analysis utilizing Pearson’s correlation is performed to examine the bivariate correlation between the study’s variables. Based on Baron and Kenny’s methodology [17], this stage is conducted to investigate the potential for a multivariate (moderating and mediating) link among the study’s variables.

Furthermore, inferential statistics using simple and multiple regression analyses were utilized to test the multivariate relationships among the dependent variable (PI), independent variables (environmental concern, subject norms, health factor, Halal safety certificate, DF, past experience factor, and price factor), mediator (personal attitude), and moderator variable (consumers’ ethnicity). Each environmental concern, subject norms, Halal safety certificate, personal attitude, and PI refers to the means sum of three items each. Each health factor, PI, and past experience factor refers to the means sum of two items. Meanwhile, the packaging design is the means sum for four items. The PROCESS v4.1 of Hayes [87] and four phases of regression analysis for Baron and Kenny [17] and Fritz et al. [88] are used to examine the mediation effect. The moderating effect is also tested using PROCESS v4.1 for Hayes [87]. Each associated factor (independent variable) was multiplied by ethnicity (moderator) to obtain the interaction term for the moderator effect. A p-value less than 0.05 is regarded as significant in the analysis. Version 23 of the statistical package for the social sciences (SPSS) is used to analyze the study’s data. Social science data can be analyzed using SPSS, a comprehensive computer application for statistical analysis.

## 4. Results

### 4.1. Sociodemographic profile of the study

The 330 participants’ demographic profile is explained in detail in the previous study by Najm et al. [41]. Female participants made up 53.6% (N = 177) of the total samples, 7.2% more than male participants. Most of the participants (N = 121, 36.7%) were in the age group of 18- to 27-year-old, followed by 27.9% between 28- to 37-year-old (N = 92); meanwhile, 12.8% were over 48-year-old (N = 42). Regarding ethnicity, 40.9% (N = 135) of the participants were Malays, followed by 36.4% (N = 120) Chinese, and 22.7% (N = 75) Indians. Most of the participants (N = 182, 55.2%) were Bachelor’s degree or equivalent holders, 23.0% were Master’s degree holders (N = 76), 12.7% were Doctoral degree holders (N = 42), 7.6% had Diploma certificates (N = 25), and only 1.5% had secondary school certificates (N = 5). Regarding occupation, most of the participants (N = 151, 45.8%) were employed, followed by 31.8% students (N = 105), 11.8% self-employed, 6.1% housepersons, and only 4.5% retirees. For monthly household income, 23.6% of the participants reported an income of RM9,001 and above, 22.7% of RM3,001 –RM6,000, and 17% of less than RM2,000.

### 4.2. Descriptive and bivariate relationship

Table 3 shows the descriptive and bivariate analysis for the study’s variables. It shows that the participants’ mean score for the ethnicity (three ethnicities) is .92 ± .806, referring to the homogeneous participants. Pearson’s correlation shows that most participants have a positive PI (2.89 ± .853) and personal attitude (3.04 ± .879) for NSCPs (2.89 ± .853). Overall, a mean value >2.5 indicates that most participants prefer purchasing NSCPs. The participants also show a positive attitude toward all the related factors (IVs) of PI (mean >2.60). Besides, the bivariate Pearson’s correlation in Table 3 shows that the participants’ ethnicity strongly correlates with PI and other related variables (p< .05).

**Table 3:** Bivariate relationship of the study variables.

Items		Mean ± SD		*(1)*	*(2)*	*(3)*	*(4)*	*(5)*	*(6)*	*(7)*	*(8)*	*(9)*	*(10)*	*(11)*	*(12)*	*(13)*	*(14)*	*(15)*
***1***	Gender	.54 ± .499	(0–1)^1^	-														
***2***	Age groups	1.19 ± 1.198	(0–4)^2^	-.022	-													
***3***	Education level	4.38 ± .857	(0–6)^3^	.000	.188**	-												
***4***	Occupation	1.85 ± 1.399	(0–4)^4^	-.147**	.628**	.174**	-											
***5***	Marital status	.52 ± .579	(0–3)^5^	.034	.591**	.197**	.429**	-										
***6***	Monthly household income	2.13 ± 1.407	(0–4)^6^	.008	.418**	.249**	.354**	.368**	-									
***7***	Ethnicity	.92 ± .806	(0–2)^7^	-.057	-.162**	.083	-.217**	-.052	.048	-								
***8***	Purchase intention (PI)	2.89 ± .853	(0–4)^8^	.010	-.054	.080	.015	-.030	.006	-.210**	-							
***9***	Personal attitude	3.04 ± .879	(0–4)^8^	.005	-.073	.163**	-.006	-.064	-.014	-.152**	.616**	-						
***10***	Environmental concern	3.05 ± .886	(0–4)^8^	.028	-.119*	.109*	-.125*	-.071	-.090	-.172**	.600**	.864**	-					
***11***	Subject norms	2.70 ± 1.06	(0–4)^8^	.030	-.127*	.075	-.143**	-.043	-.101	-.053	.273**	.375**	.351**	-				
***12***	Health factor	3.20 ± 1.02	(0–4)^8^	.012	-.190**	.137*	-.019	-.080	-.037	-.173**	.511**	.639**	.645**	.235**	-			
***13***	Halal safety certificate	2.64 ± 1.06	(0–4)^8^	-.019	-.019	.030	.027	-.031	-.202**	-.250**	.347**	.447**	.432**	.424**	.447**	-		
***14***	Packaging design	2.86 ± 1.06	(0–4)^8^	-.023	.001	.083	-.004	-.006	-.073	-.125*	.472**	.576**	.587**	.592**	.377**	.456**	-	
***15***	Past experience factor	2.55 ± 1.02	(0–4)^8^	-.030	.020	.003	.040	-.011	-.028	-.213*	.313**	.406**	.406**	.387**	.281**	.366**	.495**	-
***16***	Price factor	2.69 ± .861	(0–4)^8^	-.162**	.004	-.032	-.048	.056	-.038	-.154**	.409**	.490**	.481**	.435**	.377**	.298**	.568**	.436**

**Note:** The table reports Pearson’s correlations.

*P < 0.05 (2-tailed).

**P < 0.01 (2-tailed).

Values:

^1-7^(refer to Najm et al. 2023, Table 2);

^8^(0 = Strongly disagree; 1 = Disagree; 2 = Neither agree nor disagree; 3 = Agree; 4 = Strongly agree).

**Source:** Author’s data analysis and Najm et al. [41]

Pearson’s correlation also shows a significant linear association between PI for NSCPs and related factors (p< .05). These results indicate the possibility of a multivariate (moderating and mediating) relationship among the study’s variables. As a result, the significant variables in Pearson’s correlation analysis are included in the regression analysis to predict the moderation and mediation interaction of personal attitude and ethnicity with the related factors and PI for NSCPs. However, the bivariate analysis does not show any significant association between the sociodemographic variables see Table 3.

### 4.3. Factors affecting PI and mediating role of personal attitude on the PI model

The present study aims to determine wide-range predictors (factors) of intention to purchase NSCPs among multicultural (Malaysian) customers (Hypothesis 1 to Hypothesis 7), including ethnicity (Hypothesis 8). It also investigates the potential mediating effect of personal attitude between the independent variables (environmental concern, subject norms, health factor, Halal safety certificate, packaging design, past experience factor, and PE) and consumers’ intention to purchase NSCPs (Hypothesis 9). The four-step regression analysis using PROCESS v4.1 utilizes to test hypotheses 1–9 based on the approach of Baron and Kenny [17] and Hayes [87], see Tables 4 and 5. To determine the personal attitude mediation role on the model, setting the sample to 5,000, the confidence level to 95%, and the model to number (4) is used. The first step represents a regression analysis for the independent outcome variable (PI) with each of the seven independent variables (environmental concern, subject norms, health factor, Halal safety certificate, packaging design, past experience factor, and PE).

**Table 4:** Independent variables predict PI and personal attitude toward NSCPs.

Steps	Hypothesis	Relationship	β	*t-statistic*	p-statistic	Theory support
***Step 1*:** *Regression analysis to predict PI (dependent variable/ outcome)*		*(Constant)*			.000	
	*H1*	*EC → PI*	.577	13.576	.000***	Supported
	*H2*	*SN → PI*	.253	5.139	.000***	Supported
	*H3*	*HF → PI*	.425	10.764	.000***	Supported
	*H4*	*HSC → PI*	.279	6.690	.000***	Supported
	*H5*	*PD → PI*	.497	9.685	.000***	Supported
	*H6*	*PEF → PI*	.267	5.972	.000***	Supported
	*H7*	*PF → PI*	.404	8.109	.000***	Supported
***Step 2*:** *Regression analysis to predict PA (mediator)*		*(Constant)*			.000	
		*EC → PA*	.858	31.126	.000***	
		*SN → PI*	.359	7.315	.000***	
		*HF → PA*	.548	15.037	.000***	
		*HSC → PA*	.371	9.012	.000***	
		*PD → PA*	.627	12.763	.000***	
		*PEF → PA*	.357	8.052	.000***	
		*PA → PA*	.500	10.175	.000***	
***Step 3*:** *Regression analysis to predict PI by PA*		*(Constant)*			.000	
	*H8*	*PA → PI*	.598	14.178	.000***	Supported

**Note:** PI = purchase intention; EC = environmental concern; SN = subject norms; HF = health factor; HSC = Halal safety certificate; PD = packaging design; PEF = past experience factor; PF = price factor; PA = personal attitude toward natural skincare products.

*P< 0.05.

**P< 0.01.

***P< 0.001.

Model step one: Dependent Variable: PI. Independent Variable: related factors. R> 0.270, R^2^> 0.070, F> 26.400, P = 0.000. Model step two: Mediator Variable: PA. Independent Variable: related factors. R> 0.370, R^2^> 0.135, F> 53.700, P = 0.000. Model step three: Dependent Variable: PI. Mediator Variable: PA. R> 0.610, R2> 0.380, F> 200.000, P = 0.000.

**Source:** Author’s data analysis.

**Table 5:** Personal attitude mediation role personal attitude on the model (H9).

*Steps*	Relationships	Effect	β	*t-statistic*	p-statistic	Mediation
***Step 4*:** *Mediation effect of PA on PI and its predictors*	*EC → PA → PI*	Indirect	.323	-	.000***	Partial
		Direct	.255	3.105	.002**	
		Total	.376	4.540	.000***	
	*SN → PA → PI*	Indirect	.208	-	.002**	Full
		Direct	.046	1.045	.296	
		Total	.580	12.756	.000***	
	*HF → PA → PI*	Indirect	.261	-	.000***	Partial
		Direct	.164	3.564	.000***	
		Total	.475	8.824	.000***	
	*HSC → PA → PI*	Indirect	.208	1.837	.002**	Full
		Direct	.072	1.837	.067	
		Total	.559	11.910	.000***	
	*PD → PA → PI*	Indirect	.313	-	.000***	Partial
		Direct	.184	3.328	.001**	
		Total	.500	9.849	.000***	
	*PEF → PA → PI*	Indirect	.203	-	.003**	Full
		Direct	.064	1.583	.114	
		Total	.568	12.342	.000***	
	*PF → PA → PI*	Indirect	.265	-	.000***	Partial
		Direct	.138	2.844	.005**	
		Total	.405	8.108	.000***	

**Note:** PI = purchase intention; EC = environmental concern; SN = subject norms; HF = health factor; HSC = Halal safety certificate; PD = packaging design; PEF = past experience factor; PF = price factor; PA = personal attitude toward natural skincare products.

*P< 0.05.

**P< 0.01.

***P< 0.001.

Independent variable 1: EC-PA- PI: R = .630, R2 = .398, F = 107.972, P = .000. SN-PA-PI: R = .618, R2 = .382, F = 101.087, P = .000. HF-PA-PI: R = .635, R2 = .403, F = 110.442, P = .000. HSC-PA-PI: R = .622, R2 = .386, F = 102.919, P = .000. PD-PA-PI: R = .633, R2 = .400, F = 109.129, P = .000. PEF-PA-PI: R = .620, R2 = .385, F = 102.220, P = .000. PF-PA-PI: R = .629, R2 = .395, F = 106.726, P = .000.

**Source:** Author’s data analysis.

**Fig 2:**
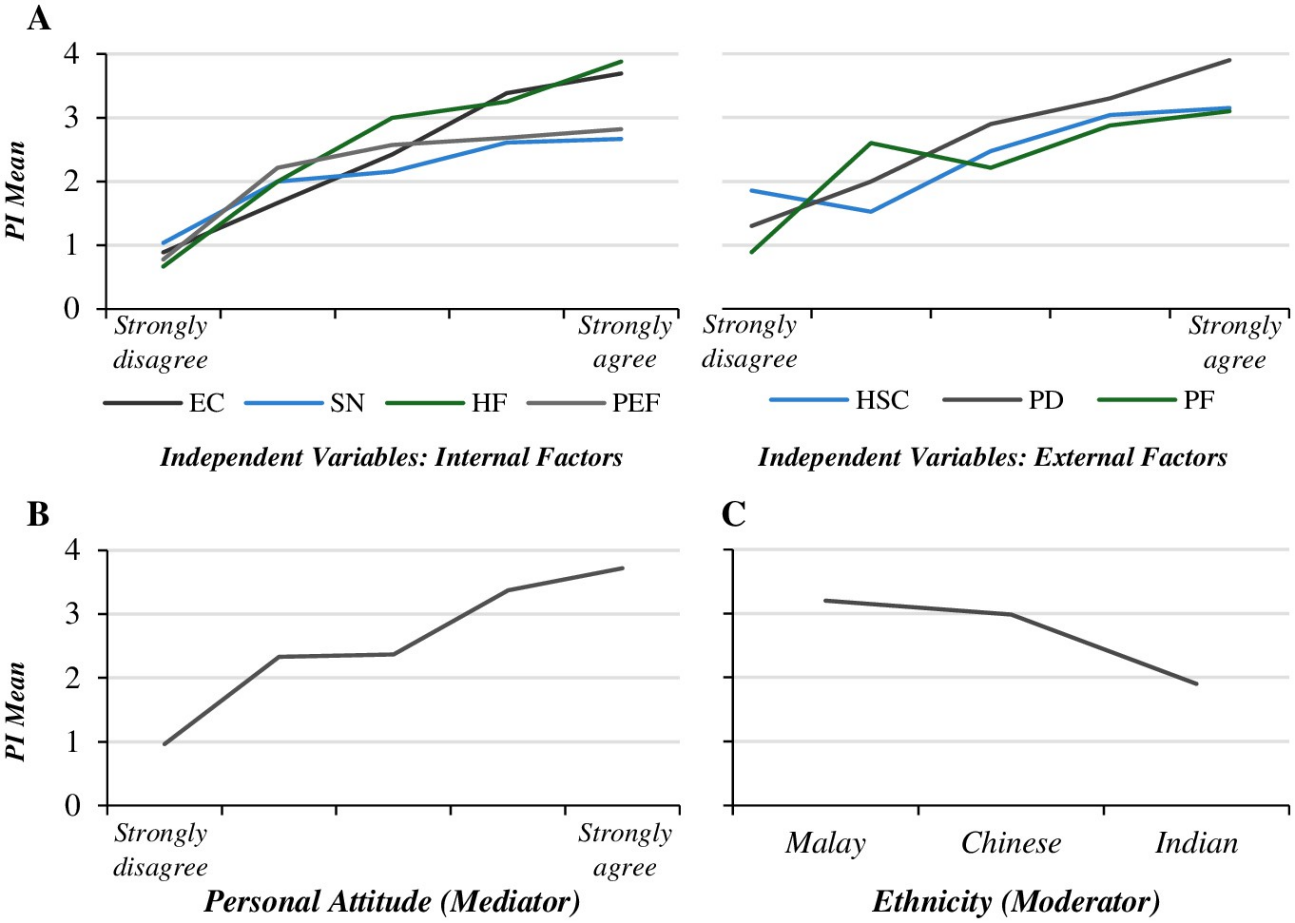
Factors (IVs) predicting PI (DV). (A) Relationship between the independent variables (internal and external factors) and the PI. (B) Relationship between the personal attitude (mediator) and the PI. (C) Relationship between ethnicity (moderator) and the PI.

The first step produces significant models with all the independent variables (R> .270, R2> .070, F> 26.400, P = .000); see Table 4. It shows a significant positive direct effect of the independent variables on the PI. Therefore, the predictors of multicultural consumers’ intention to purchase NSCPs are environmental concern, health factor, packaging design, price factor, Halal safety certificate, past experience factor, and subject norms, respectively. This result indicates the validity of hypotheses 1–7. Overall, with the higher values of environmental concern, health factor, packaging design, price factor, Halal safety certificate, past experience factor, and subject norms participants have, their PI toward NSCPs increase (see Fig 2A). Fig 2C also shows that Malay and Chinese participants have higher PI toward NSCPs than Indians.

The second step of regression analysis tests the direct relationship between each of the seven independent variables (environmental concern, subject norms, health factor, Halal safety certificate, packaging design, past experience factor, and PE) and the mediator variable (personal attitude), as shown in Table 4. The second step indicates significant models (R> .370, R2> .135, F> 53.700, P = .000) for each environmental concern, packaging design, health factor, price factor, Halal safety certificate, subject norms, and past experience factor with the personal attitude. This result indicates that with the higher values of environmental concern, packaging design, health factor, price factor, Halal safety certificate, subject norms, and past experience factor participants have, their personal attitude to natural skin care products increases. The third step includes conducting a regression analysis for the mediator variable (personal attitude) on the dependent variable (PI). This step also produced a significant model (R> .610, R2> .380, F> 200.000, P = .000). The model shows that personal attitude significantly positively affects the PI (β = .598, P = .000), indicating that participants with a high personal attitude toward natural skin care products have high PI for these products (see Fig 2B). This result also shows the validity of Hypothesis 8. These three steps of regression analysis are essential for the fourth step (mediation effect). Therefore, the significant variables in the previous three steps are included in the mediation analysis.

The fourth step included regression analysis using PROCESS v4.1 for the dependent variable (personal attitude), and independent variables, see Table 5. Furthermore, Personal attitude has a significant partial mediator mediation effect between environmental concern (P.01), health factor (P.001), packaging design (P.01), price factor (P.01), and PI, as indicated by the effects equation of the mediator. Each environmental concern, health factor, packaging design, and price factor had a significant direct and indirect impact on PI via personal attitude (mediation path). Personal attitude also plays a significant full mediator in the relationship between subject norms, Halal safety certificate, past experience factor and PI, as there is a significant indirect effect of each subject norms, Halal safety certificate, and past experience factor on PI through personal attitude (P< .01) with no direct impact (P> .05), see Table 5, which showed the validity of Hypothesis 9. Therefore, PROCESS v4.1 demonstrates that personal attitude partially mediates the relationship between the environmental concern, health factor, packaging design, price factor and PI. Personal attitude also fully mediates the relationship between the subject norms, Halal safety certificate, past experience factor and PI toward natural skin care products among multicultural consumers in Malaysia, based on Baron and Kenny approach [17].

### 4.4. Moderated mediation role of the ethnicity on the model

The present study also seeks to test the potential moderated mediation role of ethnicity (ethnic background) on the PI model (Hypothesis 10). Similarly, PROCESS v4.1 by Hayes [87] uses to test the moderated mediation effect of ethnicity on the variables of the study, using a bootstrap sample of 5,000, a confidence level of 95%, and a process model number (59).

**Fig 3:**
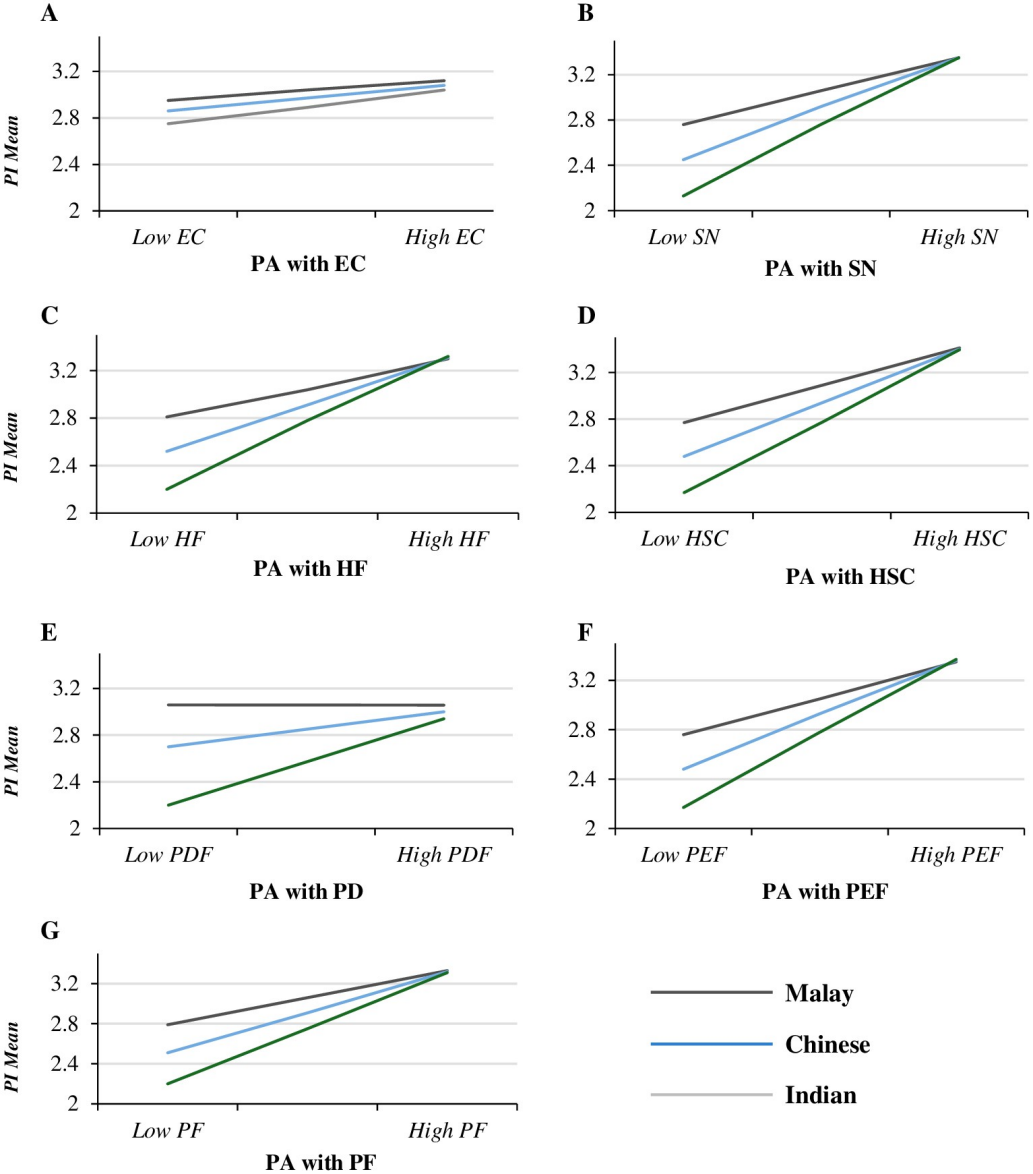
Moderated (ethnicity) mediation (PA) effect in different PI models. (A) Model: (EC → PA → PI) ← ethnicity. (B) Model: (SN → PA → PI) ← ethnicity. (C) Model: (HF → PA → PI) ← ethnicity. (D) Model: (HSC → PA → PI) ← ethnicity. (E) Model: (PD → PA → PI) ← ethnicity. (F) Model: (PEF → PA → PI) ← ethnicity. (G) Model: (PF → PA → PI) ← ethnicity. Note: PI = purchase intention; EC = environmental concern; SN = subject norms; HF = health factor; HSC = Halal safety certificate; PD = packaging design; PEF = past experience factor; PF = price factor; PA = personal attitude.

Table 6 shows that including each of the independent variables, personal attitude (mediator), ethnicity (moderator), and their interactions (IVs X ethnicity and AP X ethnicity) in the PI model led to producing significant models (R> .670, R2> .445, F> 52.900, P = 0.000). These models illustrate that including personal attitude and ethnicity in each step accounted for significant variance in PI variable. Yet, the interaction model of (environmental concern—personal attitude—PI)—ethnicity is insignificant. The effect of personal attitude as mediator is insignificant to the mean of each ethnicity, Malay to Chinese (LLCI = -.125 and ULCI = .312), Malay to Indian (LLCI = -.002 and ULCI = .238), and Indian to Chinese (LLCI = -.001 and ULCI = .205)—besides, Fig 3A shows no significant increase in the PI of participants from different ethnicities as their personal attitude toward NSCPs increased based on their environmental concern. This result means that Malay, Chinese, and Indian participants exhibited similar PI levels when their personal attitude toward NSCPs increased based on their environmental concern.

**Table 6:** Moderation role of ethnicity on the model (H10).

Relationships		Effect	p-statistics	95% Lower	95% Upper	Moderated Mediation
*Constant*			.000			Not supported
*EC X Ethnicity → PA → PI*		-	.239	-.071	.282	
*EC → PA X Ethnicity → PI*		-	.145	-.047	.315	
*(EC → PA → PI) ← Ethnicity*	Malay-Chinese	.093	.402	-.125	.312	
	Malay-Indian	.101	.054	-.002	.238	
	Indian-Chinese	.103	.051	-.001	.205	
*SN X Ethnicity → PA → PI*		-	**.174**	-.030	.165	Supported
*SN → PA X Ethnicity → PI*		-	.000***	.104	.305	
*(SN → PA → PI) ← Ethnicity*	Malay-Chinese	.338	.000***	.212	.464	
	Malay-Indian	.542	.000***	.454	.630	
	Indian-Chinese	.747	.000***	.606	.887	
*HF X Ethnicity → PA → PI*		-	**.690**	-.101	.067	Supported
*HF → Ethnicity X PA → PI*		-	.000***	.127	.345	
*(HF → PA → PI) ← Ethnicity*	Malay-Chinese	.249	.000***	.106	.393	
	Malay-Indian	.450	.000***	.348	.552	
	Indian-Chinese	.668	.000***	.531	.804	
*HSC X Ethnicity → PA → PI*		-	.031*	.009	.118	Supported
*HSC → PA X Ethnicity → PI*		-	.001**	.079	.281	
*(HSC → PA → PI) ← Ethnicity*	Malay-Chinese	.365	.000***	.233	.487	
	Malay-Indian	.518	.000***	.429	.606	
	Indian-Chinese	.683	.000***	.562	.803	
*PD X Ethnicity → PA → PI*		-	.060	-.003	.227	Supported
*PD → PA X Ethnicity → PI*		-	.005**	.050	.265	
*(PD → PA → PI) ← Ethnicity*	Malay-Chinese	.325	.000***	.195	.456	
	Malay-Indian	.459	.000***	.362	.555	
	Indian-Chinese	.604	.000***	.462	.745	
*PEF X Ethnicity → PA → PI*		-	.041*	.003	.163	Supported
*PEF → PA X Ethnicity → PI*		-	.000***	.109	.295	
*(PEF → PA → PI) ← Ethnicity*	Malay-Chinese	.331	.000***	.207	.455	
	Malay-Indian	.502	.000***	.415	.590	
	Indian-Chinese	.689	.000***	.574	.805	
*PF X Ethnicity → PA → PI*		-	.350	-.062	.175	Supported
*PE → PA X Ethnicity → PI*		-	.002**	.065	.300	
*(PE → PA → PI) ← Ethnicity*	Malay-Chinese	.304	.000***	.174	.198	
	Malay-Indian	.458	.000***	.362	.554	
	Indian-Chinese	.627	.000***	.472	.781	

**Note:** PI = purchase intention; EC = environmental concern; SN = subject norms; HF = health factor; HSC = Halal safety certificate; PD = packaging design; PEF = past experience factor; PF = price factor; PA = personal attitude toward natural skincare products.

*P< 0.05.

**P< 0.01.

***P< 0.001.

Dependent Variable: PI. Independent Variables: related internal and external factors. Mediator: PA. Moderator: Ethnicity. S-1: (EC-PA-PI)/Ethnicity: R = .671, R2 = .450, F = 52.937, P = .000. S-2: (SN-PA-PI)/Ethnicity: R = .673, R2 = .453, F = 53.636, P = .000. S-3: (HF-PA-PI)/Ethnicity: R = .678, R2 = .459, F = 55.028, P = .000. S-4: (HSC-PA-PI)/Ethnicity: R = .677, R2 = .458, F = 54.689, P = .000. S-5: (DF-PA-PI)/Ethnicity: R = .680, R2 = .462, F = 55.740, P = .000. S-6: (PEF-PA-PI)/Ethnicity: R = .676, R2 = .457, F = 54.664, P = .000. S-7: (PF-PA-PI)/Ethnicity: R = .678, R2 = .460, F = 55.339, P = .000.

**Source:** Author’s data analysis.

For the factors (subjective norms—personal attitude—PI)—ethnicity model, the mediation effect of personal attitude was significant for the mean of each ethnicity: Malay to Chinese (LLCI = .212 and ULCI = .464), Malay to Indian (LLCI = .454 and ULCI = .630), and Indian to Chinese (LLCI = .606 and ULCI = .887). The mediation effect of personal attitude is significant for the mean of each ethnicity in the (health factor—personal attitude—PI)—ethnicity model: Malay to Chinese (LLCI = .106 and ULCI = .393), Malay to Indian (LLCI = .348 and ULCI = .552), and Indian to Chinese (LLCI = .531 and ULCI = .804). Even though the interaction of subject norms X ethnicity and health factor X ethnicity is not significant: subject norms X ethnicity (LLCI = -.030 and ULCI = .165) and health factor X ethnicity (LLCI = -.101 and ULCI = .067). These results show that there is a significant moderated mediation effect in both (subject norms—personal attitude—PI)—ethnicity and (health factor—personal attitude—PI)—ethnicity models [87]. Similarly, the mediation effect of personal attitude is significant for the mean of each ethnicity in the (Halal safety certificate—personal attitude—PI)—ethnicity: Malay to Chinese (LLCI = .233 and ULCI = .487), Malay to Indian (LLCI = .429 and ULCI = .606), and Indian to Chinese (LLCI = .563 and ULCI = .803), and in the (past experience factor—personal attitude—PI)—ethnicity model: Malay to Chinese (LLCI = .207 and ULCI = .455), Malay to Indian (LLCI = .415 and ULCI = .590), and Indian to Chinese (LLCI = .574 and ULCI = .805). The interactions of Halal safety certificate X ethnicity, Halal safety certificate X personal attitude, past experience factor X ethnicity, and past experience factor X personal attitude are significant (P< .05). Therefore, there is a significant moderated mediation effect in both (Halal safety certificate—personal attitude—PI)—ethnicity and (past experience factor—personal attitude—PI)—ethnicity models.

For the (packaging design—personal attitude—PI)—ethnicity and (price factor—personal attitude—PI)—ethnicity models, the interactions of packaging design X ethnicity and price factor X ethnicity are not significant (P> 0.05). Yet, the mediation effect of personal attitude is significant for the mean of each ethnicity in the (packaging design—personal attitude—PI)—ethnicity: Malay to Chinese (LLCI = .195 and ULCI = .456), Malay to Indian (LLCI = .362 and ULCI = 555), and Indian to Chinese (LLCI = .462 and ULCI = .745), and (price factor—personal attitude—PI)—ethnicity model: Malay to Chinese (LLCI = .174 and ULCI = .198), Malay to Indian (LLCI = .362 and ULCI = 554), and Indian to Chinese (LLCI = .472 and ULCI = .781). These results show that the mediation effect of personal attitude is significantly moderated by ethnicity in both (packaging design—personal attitude—PI)—ethnicity: and (price factor—personal attitude—PI)—ethnicity models.

Fig 3 shows that as Indian participants exhibit a positive personal attitude toward NSCPs due to their subject norms, health factor, Halal safety certificate, packaging design, past experience factor, and price factor, their PI for these products sharply increased compared to Malay and Chinese participants. Similarly, as Malay participants exhibit positive personal attitude toward NSCPs due to their subject norms, health factor, Halal safety certificate, packaging design, past experience factor, and price factor, their PI for these products slightly increased compared to Indian and Chinese participants. These results demonstrated that moderated mediation significantly affects most IVs, including subject norms, health factor, Halal safety certificate, packaging design, past experience factor, and price factor, in the model. Only the mediation role of personal attitude is not moderated by ethnicity in the (environmental concern—personal attitude—PI)—ethnicity, which showed partial validity of Hypothesis 10 [87].

## 5. Discussion

The purpose of the present study was to examine the potential moderated mediation role of ethnicity in correlation between related factors and purchase intention toward NSCPs among multicultural consumers. To the authors’ knowledge, this is the first study to investigate the moderated mediation approach for PI model in a multicultural market. Existing research in this field mainly considers either the mediating effects of pro-environmental belief [24], green attitude [35] on the purchase intention, or the moderating effect of ethnicity [80, 89] and brand trust [13] on the purchase intention model. The moderated mediation model is developed in a few green marketing research based on the conceptual framework of Preacher et al. [18], such as moderated mediation of media exposure and green trust by Yang et al. [90] and safety certificates Najm et al. [41]. In the extended literature, despite the call for investigation of customer purchase intention to increase natural products purchases [35, 91], there is still a lack of studies that integrate comprehensive variables in the model of natural products purchase intention.

**Fig 4:**
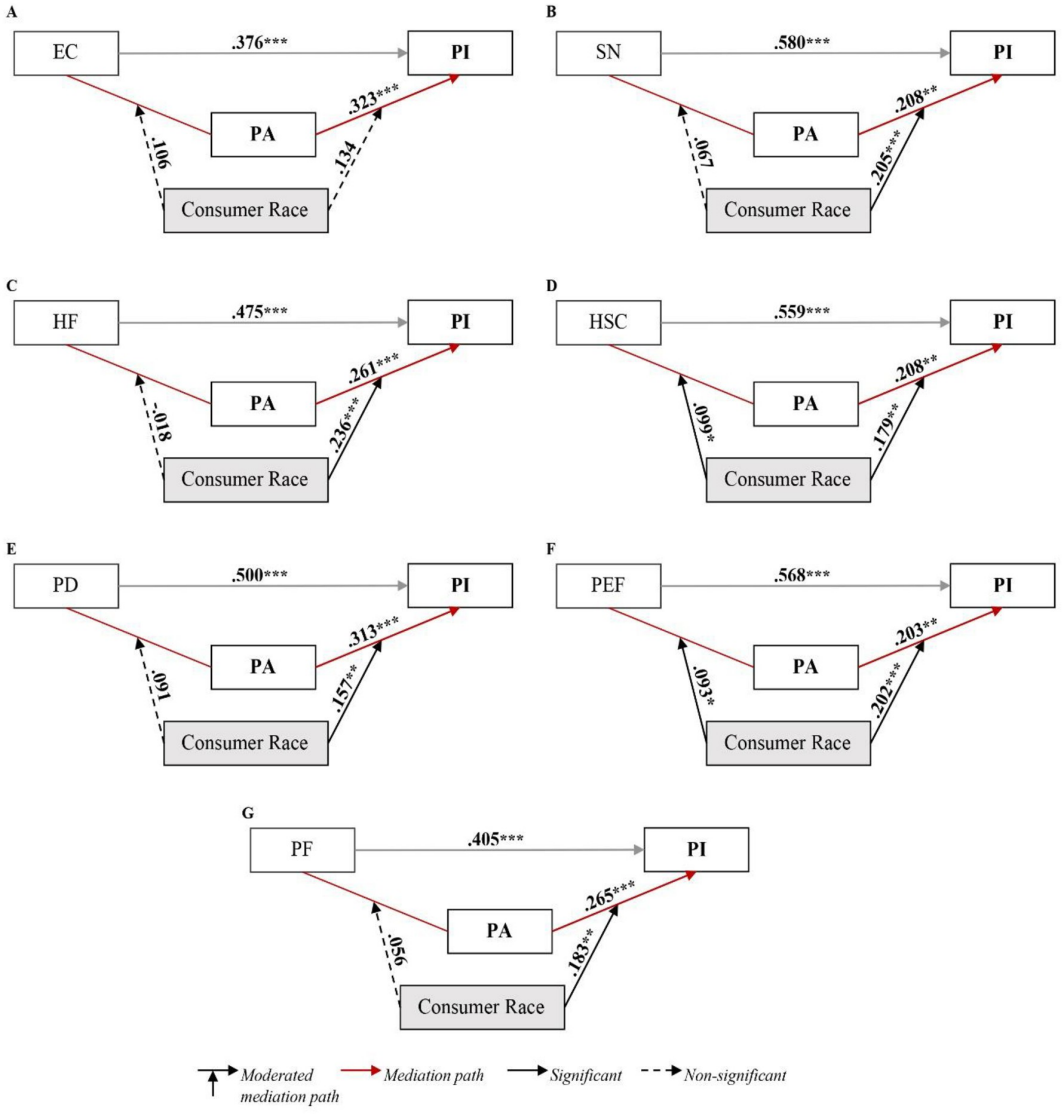
Moderated mediation path on the PI toward NSCPs model. (A) Model: (EC → PA → PI) ← Ethnicity. (B) Model: (SN → PA → PI) ← Ethnicity. (C) Model: (HF → PA → PI) ← Ethnicity. (D) Model: (HSC → PA → PI) ← Ethnicity. (E) Model: (PD → PA → PI) ← Ethnicity. (F) Model: (PEF → PA → PI) ← Ethnicity. (G) Model: (PF → PA → PI) ← Ethnicity. Note: PI = purchase intention; EC = environmental concern; SN = subject norms; HF = health factor; HSC = Halal safety certificate; PD = packaging design; PEF = past experience factor; PF = price factor; PA = personal attitude.

On the other hand, consumers’ ethnic background plays a critical role in forming their PI in the natural products market [30]. Individuals from specific ethnicities are expected to have different environmental, social, and personal beliefs from other ethnicities individuals, especially in Malaysia [28]. Therefore, ethnic differences in purchase intention based on social, cultural, and environmental beliefs are expected to be found. Thus, the present study tests the moderated mediation role of the ethnicity of multicultural (Malaysian) consumers on the natural products PI model. The outcome reveals that multicultural consumers’ ethnicity significantly moderates the personal attitude mediation in the correlation between most factors (including subject norms, health factor, Halal safety certificate, packaging design, past experience factor, and price factor) and PI toward NSCPs, see Fig 4. The finding aligns with the results from existing literature conducted by Chen et al. [92], who confirm that participants’ backgrounds are a significant moderator in the purchase intention model. However, the present study’s findings indicated that the ethnicities of multicultural consumers do not moderate the personal attitude mediation in the correlation between environmental concern and PI toward NSCPs. These results indicate the partial validity of Hypothesis 10, see Fig 4A. More specifically, the results show a positive correlation between several factors (subject norms, health concerns, Halal safety certificate concerns, concerns about packaging design, past experience concerns, and cost concerns) and PI toward NSCPs mediated by personal attitudes moderated by consumers’ ethnicities. These results contribute to findings reported by Mamun et al. and Wang et al., who confirmed that subject norms, health concerns, and packaging design significantly affect the intention to purchase skincare products through mediation factor [5, 50]. Interestingly, whenever Indian consumers exhibited a positive attitude toward NSCPs, their PI for them sharply increased due to their subject norms, health concerns, Halal safety certificate concerns, packaging design concerns, past experience concerns, and cost concerns compared to Malay and Chinese consumers. In contrast, whenever Malay consumers’ attitudes toward NSCPs increased, their PI slightly increased compared to Chinese and Indian consumers due to the mentioned factors. These findings also contribute to another Asian quantitative study that finds a possible moderator effect of consumers’ cultural factors in the product purchase model [5].

The present study also contributes to the results that Ahmad et al. [62] reported, who confirmed that knowledge of the Halal certificate increased Malaysians’ PI toward (Halal) cosmetic products. However, another study from Africa conducted by Chikazhe et al. [91] shows that sociodemographic background does not moderate the relationship in the marketing model. These different results regarding the moderator effect in the PI model might be due to the sociocultural background of Asian (Malaysian) society, which differed from that of communities in Africa. Similarly, an empirical study from China by Yang et al. [90] provides the role of consumers’ gender on the relationship between green PI and external factors. Yet, there is a shortage of research on the moderated mediation role of ethnic background in the PI model.

Furthermore, the present study investigates the possible mediating role of personal attitudes (internal factor) between several factors (internal and external) and multicultural consumers’ PI for NSCPs. The extant literature indicates the mediating effects of personal values (internal factor) on sustainable purchasing intention [16]. However, there is a shortage of evidence on the effect of personal attitude as a moderator factor in the relationship between PI and related factors in the moderated mediation model. The findings of the present study disclose the significant mediating role of multicultural consumers’ personal attitudes toward NSCPs in the PI model. The result shows that multicultural consumers’ personal attitudes partially mediate the relationship between environmental concerns, health factors, packaging design, price factors, and consumers’ PI. Multicultural consumers’ personal attitudes also fully mediate the relationship between subject norms, Halal safety certificate, past experience factor, and their PI for NSCPs. These results indicate the validity of Hypothesis 9 regarding the mediator role of personal attitude in the PI model. The results mean that multicultural consumers’ personal attitudes toward NSCPs are influenced by several internal and external factors that lead to specific purchase intention for these products. This finding complements the results of Kumar et al. [59], who test the mediation effect in the natural products PI model. However, an empirical study focused on perceived risk and trust factors for green electronics products shows the full validity of the mediation mechanism hypothesis in the green purchase model Chen et al. [92]. A possible reason for these different findings is the type of the included factors in the investigation.

Meanwhile, the regression analysis of the study shows that PI has a significant positive direct association with each factor, demonstrating the validity of Hypotheses 1 to 7. Therefore, the predictors of PI toward NSCPs among multicultural consumers include environmental concern, subject norms, Halal safety certificate, health factors, products’ packaging design, past experience, and product price. This result indicates that the deeper environmental and health concerns that multicultural consumers have, the higher the PI of NSCPs. This result aligns with Jaini et al. that environmental beliefs eventually affect consumers’ personal norms and green purchase intention [26]. External factors, such as the availability of Halal safety certificates on product packaging, packaging design, and reasonable price, positively increase PI for NSCPs, which is consistent with the findings emphasized [5, 62]. Besides, having past experience with similar products and social pressure (subject norms) increases PI to NSCPs among multicultural consumers. This result complements the evidence on the significant relationship between sustainable PI and price, social factors, and past experience factors reported by Liobikienė and Bernatonienė [33]. Yet, there is a dearth of empirical evidence that indicates the effect of the wide range of related factors on natural product PI in the multicultural market.

Furthermore, the findings also support the theoretical model of Hypothesis 8 on the significant effect of the personal attitude of multicultural consumers on their intention to purchase NSCPs. This result means that the higher the positive attitudes toward NSCPs that multicultural consumers have, the higher the PI they show toward these products, which matches the results of Mamun et al. [5] This finding is also in line with a quantitative study by Shimul et al. [15], which found that consumers’ attitude significantly impacts their PI for green cosmetics. However, the bivariate analysis of the present study does not support a significant association between the demographic variables and PI. Similarly, Chikazhe et al. [91] found no effect of demographic background (gender, age, education, and income) in the study model.

In sum, the present study highlights the natural PI and related factors in the skin care products industry and the multicultural market. The study’s theoretical model is developed based on the existing body of literature and VBN, TBP, and TCV theories under the approach of Baron and Kenny [17]. The study outcomes indicate the direct association between each independent variable, sociocultural (ethnicity) variables, and PI. Most importantly, the study supports the moderated mediation role of consumer ethnicity in the relationship between the PI of NSCPs and the subjective norms, health concerns, concerns about Halal certificates, packaging design factors, past experience factors, and price factors mediated by personal attitudes. However, ethnicity is not a moderated mediator in the correlation between the PI toward NSCPs and environmental concerns mediated by personal attitude. Thus, the study confirms that ethnicity partially moderated mediation roles in the PI model as a sociocultural factor. Therefore, the results indicate the validity of most of the hypotheses highlighted in the present study.

### 5.1. Theoretical contribution

The present study is a continuation of another study published previously by Najm et al. in the Journal of Sensory Studies [41]. The current data contribute to the previous studies by developing a new theoretical model on the moderated mediation role of consumers’ ethnicities in the PI model in the context of the natural skin care products market. It contributes to a better understanding of the moderated mediation mechanism in the PI model by utilizing ethnicity as a moderator of the path. It also determines the comprehensive factors affecting natural cosmetics purchase intention. The present study contributes to the existing body of knowledge in the fields of sustainable industry and multicultural markets by integrating social-cultural in sustainability and marketing management. This study contributes to linking several theoretical bases of the existing research, VBN, TBP, and TCV theories. Therefore, it provides a new theoretical model by proposing the relationships of sociodemographic background to the broad factors of purchase intention. Previous studies highlighted that either internal or social factors significantly affect purchase intention [5]. Specifically, the present study is one of the pioneer studies in the field that studies the moderated mediation effect of ethnic background on the relationship between a wide range of environmental, social, personal, and physical factors and PI. Ethnic background is also indicated in the previous literature as a possible factor affecting consumers’ behavior in marketing management studies [28, 51]. However, there is a shortage of literature examining the role of ethnic background (ethnicity) in the correlation between PI and related factors. Therefore, this study aims to fill the gap in the existing literature. The present study reveals the critical effect of consumers’ ethnicities on their natural skin care product PI in multicultural communities in Malaysia. It suggests that consumers’ ethnic background is essential to purchasing natural care products. Therefore, it provides a theoretical foundation for researchers, professionals, product industrialists, and marketers to consider consumer sociodemographic factors in future work and research.

### 5.2. Practical application and social contributions

The present study provides a holistic perspective on promoting sustainable consumption in multicultural markets and industries. It shows the critical role of consumers’ ethnicities and attitudes in forming their purchase intention in multicultural markets. Therefore, natural skin care product industrialists and marketers should consider the variety of consumers’ ethnic backgrounds and the factors that affect the preferences of different cultural backgrounds when promoting sustainable skin care products, especially in multicultural societies. They should also consider the common factors that positively affect different ethnicities in the multicultural (Malaysian) skincare product markets to reduce the ethnic gap. It also provides a conceptual framework for policymakers to implement the national climate change plan developed by the Ministry of Natural Resources and Environment. By highlighting numerous physical, social, and personal issues, the present study also makes contributions to the cultural and sociological aspects. It improves knowledge of the common elements influencing consumers’ intentions to buy natural skin care products from various social and cultural backgrounds. Therefore, rather than concentrating on consumers, green and social marketers are encouraged to highlight the common elements that benefit consumers from many social and cultural backgrounds. The relevance of the present study is therefore found in the connection between consumer purchase intention, sustainability, and marketing management research.

### 5.3. Limitations and future research directions

There are certain limitations to the present study. Firstly, the study sampling was restricted to convenience samples from Malaysia to represent multicultural consumers. Participants from the international markets may be considered in future studies. As a result, the conclusion could not be universally applied. Secondly, the study collected primary data using a quantitative methodology. Future studies could consider mixed or qualitative methods to comprehend the research questions better. Thirdly, the present study solely focused on the context of NSCPs; further research might reveal a wide variety of environmentally friendly cosmetics and dietary supplements. Due to the crucial role that sociodemographic backgrounds played in the sustainable marketing advances of multicultural countries, future research may also look into other sociodemographic variables in the model as moderated mediation factors, such as gender and income level.

## 6. Conclusion

Multicultural markets have become interesting topics to be considered in future research [21]. The multicultural market refers to markets that polarize consumers from more than one ethnic background. Marketing in multicultural markets is affected by several sociocultural factors. Therefore, the sustainable and natural products industry must consider these factors. On the other hand, sustainability is an increasing topic relevant to several domains of life in the context of economic, environmental, social, productivity, and the human condition. Sustainable or natural skin care products are made from natural ingredients without chemical agents or artificial coloring, making them more efficient and safer for human health and lifestyle [1, 5]. The industry of sustainable skin care products is concerned with products’ production processes, such as using natural ingredients and resources, clean technology, biodegradable, sustainable packaging, and reducing non-renewable resources, which contributes to enhancing environmental performance [10]. Recently, the annual average growth of the natural skin care products and cosmetics market has been significantly higher than conventional ones [35], especially in multicultural markets [5]. Globally, consumers are becoming more aware of consuming sustainable natural products and services.

The present study addresses three main aspects of enhancing the purchase intentions for sustainable natural skin care products among multicultural consumers. First, it determines the wide-range factors affecting purchase intention toward natural skin care products. Second, it investigates the mediation role of personal attitude in the correlation between purchase intention and each environmental, social, personal, and physical factor in the NSCPs market. Third, it investigates the moderated mediation effect of consumers’ ethnicities on the suggested model.

The results emphasize that several factors, including environmental and health concerns, subject norms, Halal safety certificate concerns, packaging design, price factor, past experience factor, personal attitude, and consumers’ ethnicities, predict intention to purchase natural skin care products among different consumers in Malaysia. Multicultural consumers’ ethnicities play a critical moderated mediation role in the correlation between PI toward natural skin care products and most related factors that are mediated by personal attitudes. The consumers’ personal attitudes also fully mediated the relationship between PI toward NSCPs and subjective norms factor, Halal safety certificate, and past experience factors. Furthermore, personal attitudes partially mediated the relationship between PI and environmental concern, health factors, packaging design, and price factors. Therefore, the results highlighted significant moderated mediation role of ethnicities in PI model in the NSCPs market and industry, especially in multicultural societies. Therefore, social and green marketers, managers, and practitioners in NSCPs markets should consider the wide range of factors influencing sustainable PI in the context of ethnic variety. Policymakers, practitioners, and marketers are advised to focus on the common factors that meet and satisfy the preference of all ethnicities in the Malaysian skin care product markets to reduce the ethnic gap in PI. Moreover, the study recommended that green marketers and policymakers should indicate and address various internal, social, and external factors in the PI model to be in line with preferences of various ethnicities in the Malaysian sustainable market.

## Supporting information

S1 Data (XLSX)

S2 Data (XLSX)

S3 Data (XLSX)
